# Modulation of gluteus medius activity reflects the potential of the muscle to meet the mechanical demands during perturbed walking

**DOI:** 10.1038/s41598-018-30139-9

**Published:** 2018-08-03

**Authors:** Maarten Afschrift, Lorenzo Pitto, Wouter Aerts, Robert van Deursen, Ilse Jonkers, Friedl De Groote

**Affiliations:** 1 Department of Movement Sciences, KU Leuven, Leuven, Belgium; 2 Department of Mechanical Engineering, KU Leuven, Leuven, Belgium; 3 School of Healthcare Sciences, Cardiff University, Cardiff, UK

## Abstract

Mediolateral stability during walking can be controlled by adjustment of foot placement. Reactive activity of gluteus medius (GM) is modulated during the gait cycle. However, the mechanisms behind the modulation are yet unclear. We measured reactive GM activity and kinematics in response to a mediolateral platform translation during different phases of the gait cycle. Forward simulations of perturbed walking were used to evaluate the isolated effect of the perturbation and the GM response on gait stability. We showed that the potential of GM to adjust lateral foot placement and prevent collisions during swing varies during the gait cycle and explains the observed modulation. The observed increase in stance, swing or combined GM activity causes an outward foot placement and therefore compensates for the loss of stability caused by a perturbation early in the gait cycle. GM activity of the swing leg in response to a platform translation late in the gait cycle counteracts foot placement, but prevents collision of the swing foot with the stance leg. This study provides insights in the neuromechanics of reactive control of gait stability and proposes a novel method to distinguish between the effect of perturbation force and reactive muscle activity on gait stability.

## Introduction

Falls in the elderly frequently occur during walking^1^, often resulting in significant injuries and loss of independence. Bipedal walking stability is actively controlled in the frontal plane^2,3^, whereas stability in the sagittal plane results to a larger extent from passive dynamics^4^. Active control of stability relies on the integration and processing of sensory information inducing muscle activation in response to a perturbation^5^. Active control of gait stability is commonly investigated by the use of sensory and mechanical perturbations to measure the corrective motor response^5–8^. This response to perturbation is quantified using changes in electromyographic and kinematic variables^5,6^.

Adjustment of medio-lateral foot placement is the main kinematic strategy for medio-lateral control of stability following a perturbation during walking^3,5,6,9^. Stepping in the direction of the perturbation, increases the width of the base of support to ensure that the margin of stability remains constant^9^. In addition, it is important to control that the swing foot does not collide with the ground or contralateral limb.

The gluteus medius (GM) is thought to play an important role in medio-lateral control of stability. Increased activity of the stance and swing leg GM has been observed in response to medio-lateral perturbations during walking^5,6,8^. Activity of the swing leg GM has been studied most frequently and is considered to be the main contributor to the stepping strategy after a medio-lateral perturbation during walking^5,6,8^. It has been suggested that activity of the swing leg GM abducts the hip and therefore controls the medio-lateral position of the foot^6^. This hypothesis is based on the positive correlation between swing leg GM activity and lateral foot placement during normal and perturbed walking^8^. Increased stance leg GM activity in response to a perturbation has been observed as well^6,8^, but the contribution to foot placement and gait stability is not well understood. Hof *et al*. hypothesized that stance leg GM activity stabilizes the hip joint and provides additional floor clearance, but does not contribute to lateral foot placement^6^.

The reactive response of both stance and swing leg GM is modulated during the gait cycle: Hof *et al*. found that the bilateral GM response magnitude to a perturbation depends on the timing of perturbation onset during the gait cycle. Simultaneously, the timing of the perturbation influences the direction of the stepping strategy. After a perturbation that causes a loss of stability in the direction of the swing leg, the foot in swing phase is positioned more outwards, hence an outward strategy^6^. After a perturbation in the opposite direction, the swing foot is positioned more in the direction of the stance leg (i.e. inward strategy)^6^.

The above suggests that both stance and swing leg GM muscles contribute to restoring stability after a medio-lateral perturbation during walking and that swing leg GM is important to adjust foot placement. Evidently, the muscle response is modulated depending on the perturbation timing during the gait cycle. However, the mechanisms behind this modulation are yet unclear. To gain insights into these mechanisms, it is important to unravel the individual contribution of stance and swing leg GM activity to the mechanics of the stepping strategy and to understand the potential of each muscle to influence stepping mechanics during the gait cycle (i.e. potential of the muscle to adjust foot placement and prevention of collision during swing). Correlations between changes in bilateral GM activity and changes in kinematics in response to the perturbation provide limited insight into these underlying mechanisms, because it is hard to separate the individual contribution of the perturbation force (passive response) and the action of individual muscles (active response) in relation to the mechanics and stability of perturbed walking.

Forward simulations with detailed models of the musculoskeletal system and corrective motor responses allow studying causal relations between muscle action and stability control. In simulation, the effect of the response of an isolated muscle on the kinematics can be computed. In addition, simulations can discriminate between the changes in kinematics caused by the perturbation and by the muscle response. Forward simulation has been successfully applied to study the contribution of muscle action to perturbed standing balance^10^, but not in perturbed walking because of the more complex musculoskeletal dynamics.

The main goal of this study was to identify the contribution of stance and swing leg GM to the control of gait stability during different phases of the gait cycle. We hypothesized that the potential of both muscles to (1) adjust lateral foot placement and (2) prevent collisions during swing varies during the gait cycle and explains the observed modulation in stance and swing leg GM activity. A combined experimental and simulation approach was used to test the hypothesis.

## Method

An experimental perturbation protocol was used to measure the kinematics and bilateral GM activity in response to a medio-lateral platform translation during different phases of the gait cycle. Muscle driven forward simulations were developed to establish causal relations between measured GM activity and walking kinematics and derived stability measures. Forward simulations of unperturbed and perturbed walking were used to quantify (1) the influence of the perturbation force on the mechanics of stability control (i.e. passive response) and (2) the isolated contribution of a single measured muscle response to the stepping strategy (i.e. active response).

### Experimental protocol

Eighteen healthy young subjects (age 21+/−2 STD) without movement disorders participated in the study. The experimental protocol was approved by and performed in accordance with the relevant guidelines and regulations of the School ethics committee of the School of Healthcare Sciences, Cardiff University. All participants gave written informed consent before participating in the study, which included consent to publish images that could lead to identification of the participant in an online open-access journal.

**Figure 1:**
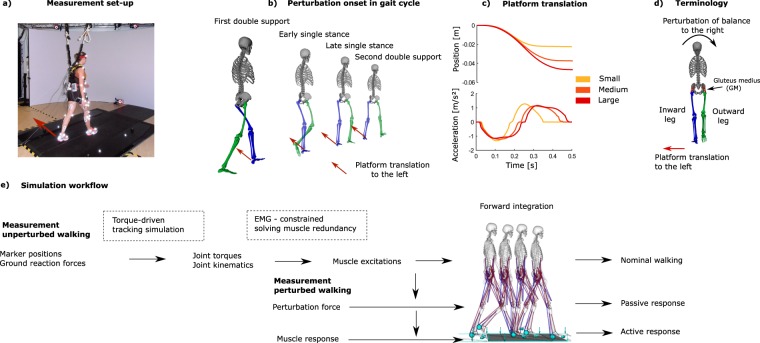
Overview measurement of the protocol and simulation method. Walking was perturbed by means of a platform translation to the left with onset at four different phases of the gait cycle (pane b) and three different amplitudes (pane c). The motion capture, ground reaction force and EMG data of unperturbed walking was used to generate a forward simulation of unperturbed walking (pane e). The platform translation was added as an external force to the forward simulation of the passive response. The platform translation and measured GM response (EMG) was added to the forward simulation of the active response.

First, the subjects walked for two minutes on a split-belt treadmill (Grail, MotekForceLink) at a fixed speed of 1.1 m/s. Second, 48 randomized unique perturbation were applied in a walking session. Each session was repeated three times with five minutes rest in between. The set of 48 unique perturbations consisted of three different magnitudes of perturbations (Fig. 1c), four directions (i.e. platform translation to the left and right, increase and decrease of the belt speed) and in four phases of the gait cycle (Fig. 1b). A gait cycle was defined as a complete left stride, starting at left heelstrike (0%) up to the next left heelstrike (100%). The perturbation was applied immediately after heelstrike (7.5% gait cycle, first double support), during early midstance (22.5% gait cycle), late midstance (37.5% gait cycle) and push-off (52.5% gait cycle, second double support).

In this study, we focused on medio-lateral stability and therefore only analyzed the response to platform translations to the left during the stance phase of the left leg. The response to a platform translation to the left during the stance phase of the left leg and the response to a platform translation to the right during the stance phase of the right leg are symmetrical. To account for this symmetry in our terminology, we will refer to the left leg by inward leg and to the right leg by outward leg based on the terminology proposed by Hof  ^6^. A platform translation to the left causes a loss of stability towards the right that is counteracted by either outward placement of the right foot, hence termed the outward leg, or inward (cross-over) placement of the left foot, hence termed the inward leg (Fig. 1d).

### Measurements and data analysis

An integrated motion capture system was used to measure the response to the perturbations. 12 Vicon T20 cameras (Vicon, Oxford) measured the trajectories of 48 retroflective markers in an extended plug in gait marker protocol^11^ at 200 Hz. Ground reaction forces were collected on a split-belt treadmill with a sampling frequency of 1000 Hz (MotekForceLink). Ground reaction forces and 3D marker coordinates were filtered with a 4^th^ order low-pass Butterworth filter with a cutoff frequency of 8 Hz. Muscle activity of the left and right GM was measured using surface electromyography (Bortec Octopus 8 channel EMG) at 1000 Hz. The EMG data was filtered with a 4^th^ order recursive Butterworth bandpass filter with cutoff frequencies of 20–400 Hz. Subsequently, the filtered signals were rectified and a linear envelope was created with a 4^th^ order recursive Butterworth low-pass filter with a cutoff frequency of 20 Hz.

Left and right heel strikes were determined from the ground reaction forces (Supplementary Section 6). The muscle responses to the perturbation were quantified by comparing the muscle activity after perturbation with the muscle activity during unperturbed walking. The processed EMG data of the last 60 gait cycles during the two minutes of unperturbed walking and the gait cycles after the perturbation were interpolated to 100 data points between two consecutive heelstrikes. The change in muscle activity in response to a perturbation was computed by subtracting the average muscle activity during unperturbed walking from the activity after perturbation. In addition, the muscle response was quantified as the time integral of the change in muscle activity during the first 300 ms after perturbation onset.

3D marker coordinates were analyzed in OpenSim^12^ to evaluate the kinematic strategy used by the subjects in response to the perturbation. A generic musculoskeletal model with 23 degrees of freedom was scaled to the subject’s anthropometry and was used to calculate joint kinematics from the recorded marker trajectories using a Kalman smoothing algorithm^13^. Stride width was computed as the average distance in the frontal plane between the left and right ankle joint center during double support. Margin of stability was computed at left and right heel strike as the distance between the extrapolated center of mass^14^ and respectively the left and right ankle joint center. A positive margin of stability indicates foot placement beyond the extrapolated center of mass in the direction of the perturbation (i.e. to the right).

### Muscle driven forward dynamic simulation of gait

A muscle driven forward simulation of unperturbed gait was created for each subject (Fig. 1e). The model was driven by 92 Hill-type muscle tendon actuators^15^. Four contact spheres were added to both feet to simulate ground reaction forces (heel, metatarsal head 1-3-5). Hunt–Crossley based contact model was used to simulate the ground contact forces, which depend on the indentation and indentation velocity of the contact spheres with respect to the ground^16^ (Supplementary Section 1). These simulations will be referred to as tracking simulations. The muscle excitations that drive the forward simulation of unperturbed walking were computed using a two-step optimization procedure. In the first step, we computed joint torques of a torque driven model that track the measured marker positions, joint torques and ground reaction forces for a representative gait cycle within the two minutes of unperturbed walking (Equation 1) by minimizing

1 $$J={\int }_{{t}_{0}}^{{t}_{end}}||{\boldsymbol{p}}-\hat{{\boldsymbol{p}}}|{|}_{2}^{2}+{w}_{1}\sum _{j}^{nF}{({{\boldsymbol{F}}}_{j}-{\hat{{\boldsymbol{F}}}}_{j})}^{2}+{w}_{2}||{\boldsymbol{T}}-\hat{{\boldsymbol{T}}}|{|}_{2}^{2}dt\,$$

where **p** is a vector containing the 3D coordinates of the markers, **F**_**j**_ is a vector containing the ground reaction forces and moments on the left and right foot (nF equals two), **T** is a vector containing the joint torques of the 23 generalized coordinates. Measured variables are labelled with the hat symbol. w_1_ and w_2_ are weights to scale the different orders of magnitude in the objective function and equal respectively 10^−7^ and 10^−5^.

In the second step, muscle excitations that generate the simulated joint torques (step 1) were computed by solving the muscle redundancy problem^17^. The objective function was the weighted sum of muscle excitations squared, a term penalizing differences between computed and measured bilateral GM activity during unperturbed walking (Equation 2, Supplementary Section 3):

2 $${J}_{2}={\int }^{}\sum _{i}^{nMuscles}{{e}_{i}}^{2}+{w}_{1}{(s{\hat{e}}_{GM}-{e}_{GM})}^{2}\,dt$$

where n = 92 is the number of muscles in the model, e_i_ is the simulated excitation of muscle i,$\,{\hat{e}}_{GM}$ is the measured activity of the GM (processed EMG), and ${e}_{GM}$ is the simulated GM excitation. *s* scales the measured excitation and is an optimization variable. Hence, we track the pattern but not the magnitude of the measured EMG signal.

The time series of simulated muscle excitation and the initial state of the model were used in a forward simulation to generate a simulated unperturbed gait pattern for every individual subject. To evaluate the influence of the perturbation and the isolated contribution of a muscle to the mechanics of perturbed walking, the influence of the perturbation (passive response) and the combined influence of perturbation and muscle activity (active response) was simulated separately for all the measured perturbation trials.

The passive response of the musculoskeletal model to the perturbation was simulated by imposing the platform translation to the muscle-driven forward simulation, controlled by the muscle excitations computed for unperturbed walking (Fig. 1e). We expressed the equations of motion in a non-inertial reference system moving with the platform. According to Newton mechanics, we therefore introduced inertial forces equal to segment mass times platform acceleration at the center of mass of each segment in the forward simulation^18^.

The active response of the musculoskeletal model to the perturbation was simulated by imposing the platform translation and adding the measured individual change in GM activity to the muscle excitations of unperturbed walking. The scaling factor (s) of the muscle redundancy solver (Equation 2) was use to scale the EMG (voltage) to the simulated muscle excitations. For every measured perturbation trial, we ran a simulation with modified GM activity of the (1) outward leg, (2) inward leg and (3) outward and inward leg.

The isolated contribution of the measured muscle activity to the mechanics of the stepping strategy was quantified by the difference between the passive and active simulated response. Since the stepping strategy depends on adequate lateral foot placement and the prevention of collisions during swing, we compared the (1) foot placement, (2) margin of stability at first heel strike after perturbation, (3) swing foot height, and (4) distance between both feet during mid-swing. The latency between the onset of the GM response and the adjustment of swing foot position was quantified as the time between perturbation onset and the time at which the difference in swing foot position between active and passive response exceeded 1 cm.

All optimal control problems (tracking simulation, muscle redundancy problem) were solved using the direct collocation software GPOPS II (Supplementary Section 2).

### Outcome measures and statistical analysis

Two-way repeated measures analysis of variance (rmANOVA) was used to evaluate the main effect of perturbation onset timing (four levels) and perturbation magnitude (three levels) on measured stride width, margin of stability and bilateral GM activity. Post-hoc pairwise comparison was used to evaluate the response after perturbation compared to unperturbed walking using Tukey’s Honest Significant Difference to correct for multiple comparisons. Shapiro–Wilk test was used to check the normality of the data. Linear regression (including Pearson’s correlation coefficient) was used to compare changes in bilateral GM activity and changes in simulated stride width and margin of stability at the first heel strike after perturbation and foot height and inter-feet distance during the first mid-swing after perturbation. Correlation between the aforementioned outcome variables and the muscle response was reported by adjusted R squared values. The slope and intercept of the linear regression was computed for the different onset timings of the perturbation (Fig. 1b). A two-sided confidence interval with an alpha level of 0.05 was used to define significance for all statistical tests.

### Data availability

The dataset and simulation software generated during the current study are available from the corresponding author on request.

## Results

### Experimental results

**Figure 2:**
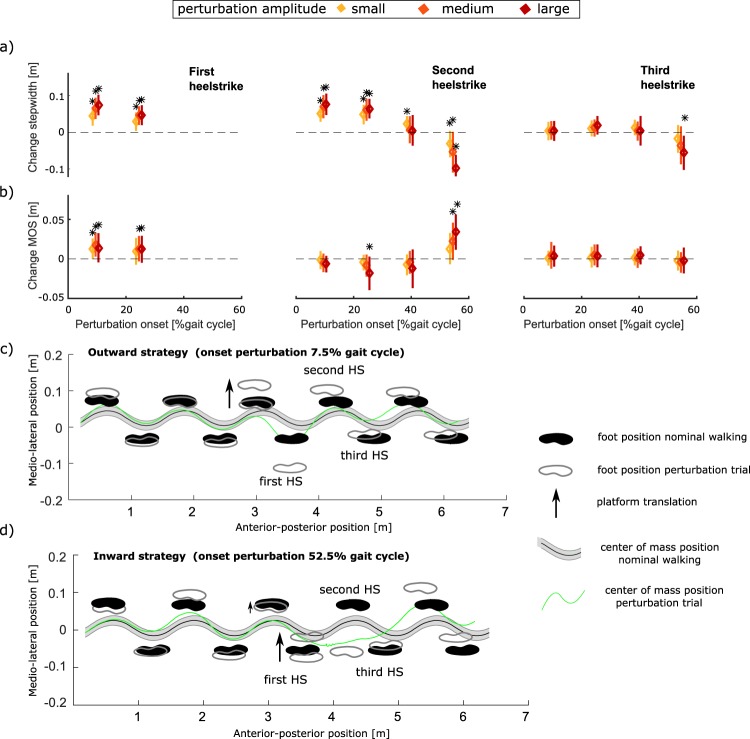
Modulation of stepping strategy in response to platform translation to the left during the gait cycle. Group mean +/- standard deviation of change in step width (**a**) and margin of stability (**b**) at the first three heelstrikes after perturbation onset compared to unperturbed walking as a function of the onset timing of the platform translation. The stars indicate results significantly different from zero (p < 0.05). An outward stepping strategy was used in response to the platform translation during first double support (**c**) (perturbation onset 7.5% gait cycle). An inward stepping strategy was used in response to the platform translation with onset during second double support (**d**) (perturbation onset 52.5% gait cycle).

The onset timing of the perturbation had a significant effect on stride width (F=59.82; p < 0.001) and margin of stability (F = 23.20; p < 0.001) at first heelstrike after perturbation. An outward strategy, characterized by an increase in stride width, was observed when the platform translation started early in the gait cycle (7.5% and 22.5% gait cycle). For this timing of perturbation, the outward leg is in swing phase and therefore gait stability can be adjusted by outward foot placement (Fig. 2c). An inward stepping strategy, characterized by a decrease in stride width, was observed when the perturbation started during second double support (i.e. 52.5% of the gait cycle) (Fig. 2d). For this timing of perturbation, the outward leg is in stance phase and therefore stability can be restored by inward foot placement of the swing leg (Fig. 2d).

**Figure 3:**
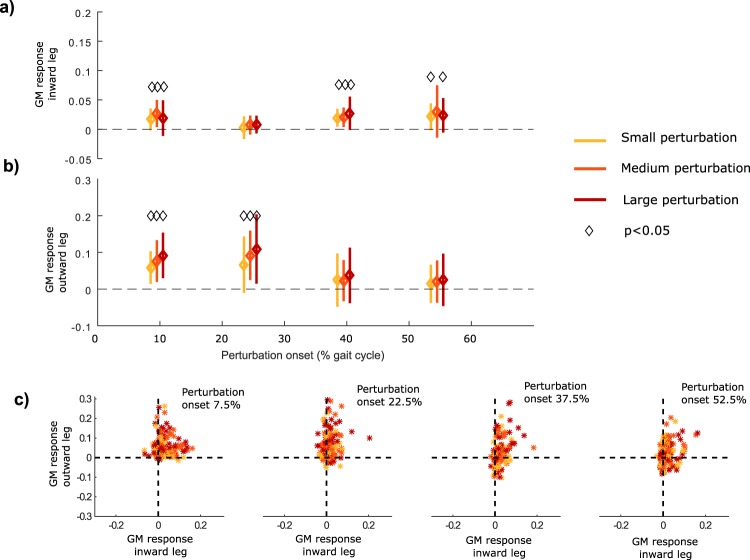
Modulation of bilateral GM activity in response to platform translation during the gait cycle. Group mean +/- standard deviation of the GM response of the inward (**a**) and outward leg (**b**) to the platform translation as a function of onset timing of the perturbation during the gait cycle. GM response significantly different from zero (i.e. unperturbed walking) is highlighted with a square (p < 0.05). The relative change in GM activity of the inward versus outward leg in response to the perturbation of all perturbation trials for the four different timings of perturbation (**c**).

**Figure 4:**
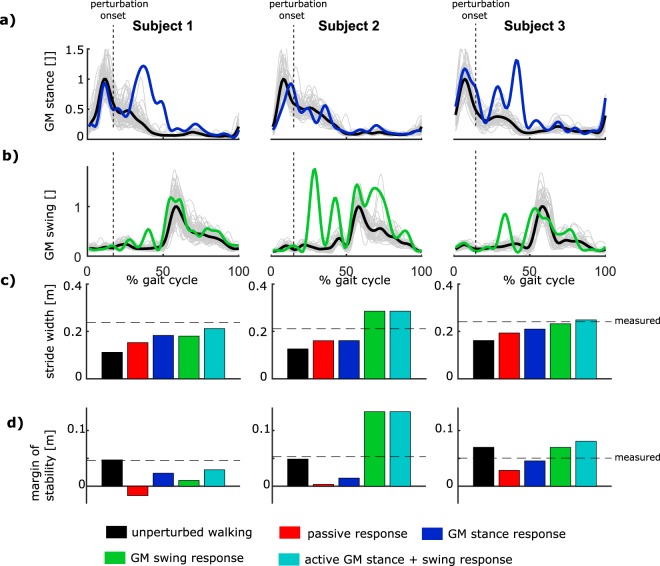
Simulated response to perturbation during first double support (7.5% gait cycle) for subjects using the GM activity of the inward (stance) leg, outward (swing) leg and a combination of both. Simulated change in stride width and margin of stability for a subjects using mainly the stance leg GM (subject 1), the swing leg GM (subjects 2) or a combination of both (subject 3). The average measured muscle activity of the stance (**a**) and swing (**b**) leg GM during unperturbed walking is shown in black (all unperturbed gait cycles in gray) and after perturbation in blue for the stance leg and green for the swing leg. The vertical black dotted line represents perturbation onset. The simulated margin of stability at first heelstrike after perturbation is shown for unperturbed walking (black), passive response (red), active response stance leg GM (blue), active response swing leg GM (green) and combined response of stance and swing leg GM (cyan).

The timing of the perturbation had a significant effect on GM activity in the inward [F = 11.47, p < 0.001] and outward leg [F = 9.55, p < 0.001] (Fig. 3). A significant increase in GM activity of both the outward and inward leg was found in response to perturbations during first double support (7.5% gait cycle, Fig. 3a). The bilateral GM response to perturbation during first double support differed substantially between subjects (Fig. 3c), although the kinematic response of the outward stepping strategy was similar (Fig. 2). The bilateral GM response was characterized by an increased GM activity of the inward, outward or a combination of both legs (Fig. 4). Muscle responses to perturbations during midstance and push-off were similar between subjects (Fig. 3c). Perturbations with onset at 22.5% of the gait cycle, when the inward leg is in early midstance and the outward leg is in midswing, caused a significant increase in GM activity of the outward leg (Fig. 3a). Perturbations with onset at 37.5% and 52.5% of the gait cycle, when the inward leg transitions from stance to swing and the outward leg is in late swing or early stance, caused an increase in GM activity of the inward leg (Fig. 3b).

An increased perturbation amplitude significantly increased stride width [F = 5.1, p = 0.006], but not the margin of stability [F = 0.57, p = 0.56] and GM activity of the inward [F = 1.31, p = 0.26] and outward leg [F = 2.55, p = 0.08] leg at first heel strike after perturbation.

### Simulation results

The simulations of unperturbed walking were in agreement with the experimental data. The root-mean square error of the tracking simulation of unperturbed walking was 0.03 cm for the marker positions, 29 N for the ground reaction forces and 6.7 Nm for the joint torques (For details, Supplementary Figs 2 and 3).

**Figure 5:**
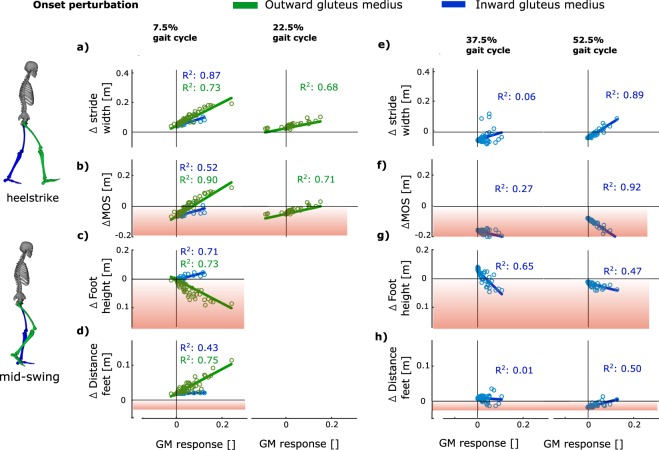
Influence of bilateral GM activity on stability and swing foot position as a function of perturbation onset timing. Linear regression between (**a**) stride width, (**b**) margin of stability, (**c**) foot height and (**d**) inter-feet distance during mid swing and GM activity of the inward (blue line) and outward leg (green line). The intercept of each regression shows the effect of the perturbation on the outcome variable without an active muscle response (i.e. passive response simulation).The slope of the regression shows the potential of the reactive muscle activity (i.e. active response) to adjust the four outcome variables. The stride width and margin of stability was evaluated at first heelstrike of the outward leg for the perturbations with onset at 7.5% and 22.5% of the gait cycle, and at first heelstrike of the inward leg for the perturbations with onset at 37.5% and 52.5% (i.e. since the outward leg is already on the ground). The red areas indicate when there is a potential loss of stability: (**b**,**f**) negative margin of stability (**c**,**g**) ground-foot collision during midswing (**d**,**h**) inter-feet collision during mid swing. The regression was only computed when a significant GM response of the inward or outward leg was observed (Fig. 3).

When adding the perturbation force to the simulation of unperturbed walking, without reactive muscle activity, we found that the perturbation had a destabilizing effect. The influence of the perturbation force on foot placement, margin of stability and collisions was visualized in Fig. 5 as the intercept of the linear regression with the vertical axis. First, a decrease in margin of stability was found in the passive response simulation for all onset timings of the perturbation. The negative intercept for margin of stability for all perturbations timings (Fig. 5b–f) shows that the perturbation caused a loss of stability without active muscle response. Second, the passive effect of the perturbation force also influenced the foot position at heel strike (Fig. 5). Stride width increased for perturbations during first double support and early single stance (Fig. 5a, positive intercept), but decreased for perturbation during late single stance and second double support (Fig. 5e, negative intercept). Third, inter-limb distance and foot height at mid-swing decreased for perturbation during second double support, which may cause inter-limb and ground-limb collision (Fig. 5g,h, negative intercept perturbation at 52.5% gait cycle).

When adding the perturbation force and the active response of the inward or outward GM to the forward simulation, we found that GM activity of both the inward and outward leg increased gait stability. However, the effect of the GM response on stride width, the margin of stability at heelstrike and inter-feet distance during mid swing varied during the gait cycle.

For perturbations with onset during first double support (7.5% of the gait cycle), when the outward leg is in swing and outward foot placement can be adjusted, the active response of the GM of both the inward and outward leg increased gait stability (Figs 4 and 5). The significant positive correlation between (1) swing leg GM response, (2) stance leg GM response, and (3) stride width and margin of stability shows that activation of both muscles increased stride width and margin of stability at first heel strike after the perturbation (Fig. 5a,b), which is visualized by the positive slope of the linear regression in Fig. 5. The steeper slope of the regression between outward GM activity and simulated stride width and margin of stability compared to the slope of the inward GM activity shows the stance leg GM has an greater potential to increase stride width (Fig. 5a) and margin of stability (Fig. 5b) for perturbations with onset during first double support. The adjustment of swing foot position, and increase in stride width, was significantly slower following activation of the stance leg GM (latency: 0.36 s +/−0.03 STD) compared to the swing leg GM (latency: 0.26 s +/−0.03 STD) (p < 0.001). In addition, the negative slope of the regression between outward GM activity and foot height shows that reactive swing leg GM activity decreased foot height during mid swing (Fig. 5c), whereas stance leg GM activity increased foot height (Fig. 5c, positive slope inward GM).

For perturbations with onset during early single stance (22.5% of the gait cycle), when the outward leg is in swing, the active response of the GM activity of the outward leg and not inward leg increased gait stability. The positive slope of the regression between outward GM activity, stride width and margin of stability shows that reactive outward GM activity increased step width and margin of stability. No active response of the inward GM was measured for this timing of perturbation and therefore did not contribute to gait stability (Fig. 3).

For perturbations with onset during second double support (52.5% of the gait cycle), when the inward leg transitions from stance to swing, the measured increase in GM activity of the inward leg prevented inter-limb collision but decreased the margin of stability. The negative slope of the regression between inward GM activity and margin of stability shows inward GM activity reduced the margin of stability (Fig. 5f). The positive slope of the regression between inward GM activity and inter-feet distance shows that reactive GM activity increased inter-feet distance during mid swing (Fig. 5h, positive slope) and therefore prevents inter-limb collision caused by the perturbation force (Fig. 5h, negative intercept). No active response of the outward GM was measured for this timing of perturbation and therefore did not contribute to gait stability (Fig. 3).

## Discussion

We quantified the effect of bilateral GM activity in response to lateral platform translations on the mechanics of reactive control of gait stability. In accordance with previously published results^6^, we found that reactive GM activity and stepping strategy are modulated during the gait cycle. In simulation, we confirmed the hypothesis that the potential of the GM of the inward and outward leg to adjust lateral foot placement and to prevent collisions during swing varies during the gait cycle. This explains the observed modulation in bilateral GM activity.

The measurements of perturbed walking showed that reactive GM activity and stepping strategy are modulated during the gait cycle^6^. The onset timing perturbation had a significant effect on the kinematics and reactive bilateral GM activity. The kinematic strategy shifted from an outward to an inward stepping strategy when the onset timing of the perturbation was later in the gait cycle (Fig. 2). Lateral translations of the stance leg with onset during first double support resulted in an outward stepping strategy, characterised by an increase in stride width and an increase in GM activity of both the inward and outward leg. Perturbations during second double support resulted in an inward stepping strategy, characterised by a decrease in stride width and increased GM activity of the inward leg.

Results from the simulated passive response showed that the platform translation perturbed stability during all phases of the gait cycle and that an active response is needed to control gait stability. All perturbations caused a decrease in margin of stability (Fig. 5, negative intercept MOS). In addition, the mechanical demands imposed by the perturbation change during the gait cycle, because the inward and outward leg transition between stance and swing phase. For perturbation early in the gait cycle, lateral foot placement of the outward leg is needed to compensate for the instability caused by the perturbation (i.e. outward stepping^9^). For perturbations late in the gait cycle, the outward leg is in stance phase and medial foot placement of the inward leg is therefore needed to prevent a fall (i.e. inward stepping^9^). In addition, perturbations during second double support in the absence of active muscle responses reduce inter-feet distance, which may cause inter-limb collision. The changes in mechanical demands imposed by the perturbation suggest that modulation of reactive muscle activity is needed to control balance.

**Figure 6:**
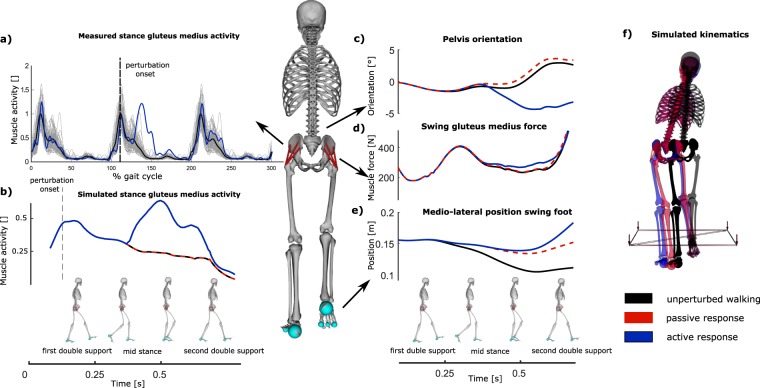
Medio-lateral foot placement controlled by stance leg GM (GM) activity in perturbations with onset during first double support. Stance leg GM activity was increased after perturbation (blue) compared to unperturbed walking (gray) (**a**). The isolated effect of stance leg GM response on walking kinematics was evaluated by comparing three forward simulations; (1) unperturbed walking (black), (2) passive response (red) and (3) active response (blue). The increase stance leg GM activity (**b**) and force tilts the pelvis upwards in the frontal plane (**c**) which stretches the swing leg hip abductors and therefore increases the swing leg GM force for the same activation (**d**). The resulting increase in swing leg hip abduction moment rotates the swing leg outwards and therefore increases the stride width (**e**). The resulting increase in stride width at heelstrike is shown in pane f.

In simulation, we showed that the potential of the inward and outward leg GM to increase the margin of stability and to prevent collisions varies during the gait cycle (Fig. 5, slope regression). Comparison between the simulated passive and active response shows the isolated effect of GM activity on the kinematics of gait stability. Our simulation results suggest that the potential of the muscle to control stability is different in stance versus swing phase and for the outward versus inward leg. Indeed, as hypothesized by Hof *et al*.^6,8^, reactive swing leg GM activity abducts the hip during swing phase and therefore controls outward position of the foot, which increases inter-feet distance during mid swing and stride width at heelstrike. This is true for both the outward and inward leg during their respective swing phases but more outward foot placement increases stability in the case of the outward leg (in line with outward stepping strategy) and decreases stability in case of the inward leg (counteracting inward stepping strategy). The contribution of the stance leg GM response to the stride width and margin of stability is more complex (Fig. 6). Reactive GM activity of the stance has been observed by^5,6,8^, but its contribution to gait stability was unknown. In simulation, we showed that an isolated increase in activity of the stance leg GM increases the stance leg hip abduction moment. The increased hip abduction moment tilts the pelvis upwards and increases the swing leg hip adduction angle (Fig. 6c). Consequently, the swing leg hip abductor muscles (i.e. contralateral GM) stretch and generate more force for the same activation due to the altered position on the force length and force velocity relation of the Hill type muscle, therefore additionally increasing the swing leg hip abduction moment (Fig. 6d). Through this mechanism, activity of the stance leg GM is mechanically coupled to pelvis and swing leg orientation, and supports lateral foot placement (Fig. 6e).

Our simulations demonstrated changes in mechanical demands imposed by the perturbation and the changing potential of the GM to control stability throughout the gait cycle, suggesting that modulation of reactive GM activity is needed to control gait stability. Indeed, the experimentally observed changes GM responses and kinematics during the gait cycle shows that the subjects modulated reactive GM activity. Hence, the combination of the experimentally observed GM responses with simulation results show that reactive GM activity was modulated during the gait cycle to compensate for the changes in mechanical demands imposed by the perturbation and changes in muscle potential to control stability throughout the gait cycle.

During first double support (i.e. 7.5% gait cycle), inward and outward GM are agonists in the control of stride width and inter-feet distance and considerable inter-subject variability in reactive GM activity was observed. Using forward simulations of the active response, we found that the isolated response of both inward and outward GM resulted in more lateral placement of the outward foot and an increased margin of stability at first heel strike after the perturbation (Fig. 5). This is in agreement with the measured increase in stride width. The measured inter-subject variability in reactive GM activity of the inward (stance) versus outward (swing) leg suggests that subjects use the redundancy in strategies, due to the agonistic function of bilateral GM, differently. On the one hand, the swing leg muscle has a higher potential to increase stride width (Fig. 5b, slope linear regression) and has a smaller delay between activity and adjustment of swing foot position compared to the stance leg GM. The swing leg GM response may therefore be a safer strategy to control foot placement. On the other hand, the response of the swing leg GM decreases foot height, and therefore requires activity of other muscles to increase foot height and prevent ground contact during mid swing. Therefore, use of stance leg GM might reduce the need to coordinate multiple muscles, thereby possibly simplifying control. Hence, the between-subjects variability in the use of stance versus swing leg GM for perturbations early in the gait cycle might reflect a trade-off between efficient foot placement and the need to coordinate multiple muscles.

For perturbations during early single stance when the inward leg is in stance and the outward leg is in swing, GM response of the outward leg and not the inward leg contributes to gait stability. The absence of stance leg GM is probably due to the larger time delay between reactive muscle activity and adjustment of swing foot position for the stance leg GM strategy compared to the swing leg GM strategy. For perturbations with onset during early single stance when the outward leg already transitioned into swing, a reactive response of the stance leg GM might be too slow to adjust contralateral swing foot position sufficiently before heel strike. In response to perturbations, when solely the swing leg GM has the potential to control gait stability, only swing leg and not stance leg GM is used.

For perturbations during second double support, the observed GM activity of the inward leg is needed to prevent inter-limb collision, although it counteracts inward foot placement. Indeed, the measured decrease in stride width and increase in margin of stability reflects a successful inward strategy when perturbation onset was at 52.5% of the stride (Fig. 2b–d). The significant increase in the GM activity of the inward leg, which transitions from stance to swing, suggests that this muscle contributes to the inward strategy. However, in simulation the increase in muscle activity caused an increase in stride width and a decrease in margin of stability (Fig. 5e,f), therefore counteracting the measured inward stepping strategy. Differences between the simulated passive and active response to the perturbation suggest that the main function of the reactive GM activity of the inward leg is to prevent collision of the swing foot with the stance leg during mid-swing (Fig. 5h, negative intercept perturbation at 52.5%).

Several limitations should be considered when interpreting these results. First, the simulations of unperturbed walking did not exactly track measured marker positions and ground reaction forces. The errors originated from dynamic inconsistencies in the measured kinematics and external forces due to measurement and modelling errors. These errors can mainly be attributed to soft tissue artefacts, simplified representation of the trunk and head by a single rigid body, and inaccuracies in the foot-ground contact model. However, tracking errors were small and we therefore suggest that they do not influence our conclusions. Second, EMG signals only give qualitative information about muscle activity and therefore the method used to scale EMG signals might have influenced the estimated magnitude of GM muscle activity. Since we used the EMG-based estimates of GM activity to drive these forward simulations, scaling inaccuracies will have influenced the reactive muscle force and therefore the simulated change in stride width, foot height and margin of stability. However, EMG scaling inaccuracies only affect the magnitude of the stepping strategy but not the relation (i.e. slope of the regression) between reactive muscle activity and the outcome variables. Therefore, the main conclusions are not sensitive to EMG scaling errors (Fig. 5). Third, simulations are sensitive to modeling choices and model parameters. However, results from a sensitivity analysis showed that the modelling assumptions had only a small influence on the simulated stride width and margin of stability and did therefore not influence the trends between changes in reactive GM response, stride width and margin of stability after perturbation (Supplementary Section 5)

The simulation method introduced in this paper has several advantages over induced acceleration analysis, which has been predominantly used to quantify the contribution of an isolated muscle to walking kinematics^19^. The present forward simulation method evaluates the accumulated effect of a perturbation and reactive muscle response on kinematic variable, whereas induced acceleration analysis evaluates the instantaneous contribution of a muscle to the measured kinematics.

## Conclusion

In conclusion, this study showed that the modulation of reactive bilateral GM activity in response to a medio-lateral perturbation of walking during the gait cycle reflects the phase dependent muscle potential to control the margin of stability and prevent inter-limb collision. Using forward simulations, it was possible to establish causal relations between the observed GM response to a perturbation and kinematic strategies for control of gait stability. First, these results showed that an increase in stance or swing leg GM activity causes an outward foot placement and therefore compensates for the stability loss caused by a lateral translation of the stance leg early in the gait cycle. Second, GM activity of the inward leg in response to a platform translation late in the gait cycle (i.e. during push-off) prevents collision between the swing foot and stance leg, although it counteracts inward foot placement. This study provides important insights in the muscle coordination of reactive control of stability during walking and proposes a novel method to quantify the contribution of the external perturbation force and the active muscle response to gait stability.

## Electronic supplementary material

Appendix

## References

[1] Berg WP, Alessio HM, Mills EM, Tong C (1997). Circumstances and consequences of falls in independent community-dwelling older adults. Age Ageing.

[2] O’Connor SM, Kuo AD (2009). Direction-Dependent Control of Balance During Walking and Standing. J. Neurophysiol..

[3] Collins SH, Kuo AD (2013). Two independent contributions to step variability during over-ground human walking. PLoS One.

[4] McGeer T, McGeer (1990). *Passive dynamic walking*. The International Journal of Robotics Research.

[5] Stokes, H. E., Thompson, J. D. & Franz, J. R. The Neuromuscular Origins of Kinematic Variability during Perturbed Walking. *Sci*. *Rep*. **7**, (2017). 10.1038/s41598-017-00942-x PMC5429788 28400615

[6] Hof AL, Duysens J (2013). Responses of human hip abductor muscles to lateral balance perturbations during walking. Exp. Brain Res..

[7] Hof AL, Duysens J (2018). Responses of human ankle muscles to mediolateral balance perturbations during walking. Hum. Mov. Sci..

[8] Rankin BL, Buffo SK, Dean JC (2014). A neuromechanical strategy for mediolateral foot placement in walking humans. J. Neurophysiol..

[9] Hof aL, Vermerris SM, Gjaltema Wa (2010). Balance responses to lateral perturbations in human treadmill walking. J. Exp. Biol..

[10] Welch TDJ, Ting LH (2007). A Feedback Model Reproduces Muscle Activity During Human Postural Responses to Support-Surface Translations. J Neurophysiol.

[11] Davis Iii RB, Õunpuu S, Tyburski D, Gage JR (1991). A gait analysis data collection and reduction technique. Human Movement Science.

[12] Delp SL (2007). OpenSim: open-source software to create and analyze dynamic simulations of movement. IEEE Trans. Biomed. Eng..

[13] De Groote F, De Laet T, Jonkers I, De Schutter J (2008). Kalman smoothing improves the estimation of joint kinematics and kinetics in marker-based human gait analysis. J. Biomech..

[14] Hof AL (2007). The equations of motion for a standing human reveal three mechanisms for balance. J. Biomech..

[15] Delp SL (1990). An interactive graphics-based model of the lower extremity to study orthopaedic surgical procedures. IEEE Trans. Biomed. Eng..

[16] Sherman MA, Seth A, Delp SL (2011). Simbody: Multibody dynamics for biomedical research. In Procedia IUTAM.

[17] De Groote F, Kinney AL, Rao AV, Fregly BJ (2016). Evaluation of Direct Collocation Optimal Control Problem Formulations for Solving the Muscle Redundancy Problem. Ann. Biomed. Eng..

[18] Kim S, Atkeson CG, Park S (2012). Perturbation-dependent selection of postural feedback gain and its scaling. J. Biomech..

[19] Neptune RR, Kautz Sa, Zajac FE (2001). Contributions of the individual ankle plantar flexors to support, forward progression and swing initiation during walking. J. Biomech..

