# Supplementary material for: Modulation of gluteus medius activity reflects the potential of the muscle to meet the mechanical demands during perturbed walking
Source: Sci Rep. 2018 Aug 3;8:11675. doi: 10.1038/s41598-018-30139-9 (PMC6076241; doi:10.1038/s41598-018-30139-9)
Supplement: Supplementary file 1 — Appendix [file 41598_2018_30139_MOESM1_ESM.pdf]

Supplementary Information: Modulation of gluteus medius activity reflects the potential of the muscle to meet the mechanical demands during perturbed walking

M. Afschrift,L. Pitto, W. Aerts, R. van Deursen, I. Jonkers, F. De Groote

Contents

1 Musculoskeletal model **1**
1.1 Passive forces 1
1.2 Muscle moment arms 2
1.3 Ground contact model 2

2 Tracking skeletal dynamics **3**
2.1 Problem formulation 4
2.2 Summary optimal control problem 4
2.3 Implementation 5
2.4 Tracking results 5

3 Tracking muscle dynamics **5**

4 Forward simulation **6**

5 Sensitivity analysis **7**
5.1 Stiffness contact model 7
5.2 Lower bounds on activity 8

6 Measurements **8**
6.1 Heelstrike detection 8
6.2 Marker protocol 10

1 Musculoskeletal model

OpenSim's gait2392 model was used to simulate musculoskeletal dynamics during walking. The model was scaled to the anthropometry and mass of the subjects based on the anatomical marker positions and ground reaction forces during a static trial (i.e. subjects is standing upright). As described below, several adjustments were made to the model.

1.1 Passive forces

Passive joint moments were added to the ankle, knee, hip and lumbar joints to simulate ligament forces. Passive joint torques were modeled as coordinate limit forces in OpenSim (Equation 1). Coefficients were selected for each joint and were based on [1] (Table 1).

$$P = k_1 e^{k_2(q-\theta_2)} + k_3 e^{k_4(q-\theta_1)} - c\dot{q} \quad (1)$$

where P is the passive joint torque, q is the joint angle and $\dot{q}$ is the angular velocity.

	k_1 $[Nm]$	k_2 $[-]$	k_3 $[Nm]$	k_4 $[-]$	$\theta_1[rad]$	$\theta_2[rad]$	c $[Nms]$
ankle PF	-12	15	11.03	-11.33	2.4	0.13	0.025
knee	-12	15	4	-15	0.74	0.52	0.025
hip flexion	5	5.05	3	-10	-0.47	1.81	0.025
hip adduction	-5	15	5	-15	-0.52	0.26	0.025
hip rotation	-5	15	5	-15	-0.52	0.52	0.025
lumbar flexion	-5	30.7	5	-20	0.52	0.17	0.025
lumbar adduction	-5	20.36	5	-20.36	-0.35	0.35	0.025
lumbar rotation	-5	20.36	5	-20.36	-0.35	0.35	0.025

Supplementary Table 1: Coefficients passive joint torques

1.2 Muscle moment arms

Computation speed in the forward simulations was increased by approximating muscle tendon length - joint angle relationships with polynomial functions. Coefficients were estimated by minimzing the difference between the muscle tendon length computed with the polynomial approximation an OpenSim's Muscle Analysis Tool. The order of the polynomial was increased stepwise until the difference between the muscle tendon length and moment arms computed with the polynomial approximation and OpenSim's Muscle Analysis Tool was smaller than 1.5 mm for the kinematics observed during normal and perturbed during walking [6].
Moment arms were computed as the partial derivative of the muscle-tendon length to the selected degree of freedom (equation 2).

$$dM_j = \frac{\partial L_{MT}}{\partial q_j} \quad (2)$$

where dM_j is the moment arm of the muscle for the joint angle q_j, L_{MT} is the muscle tendon length. Muscle tendon velocity was computed as the product of the moment arm and the angular velocity (Equation 3)

$$V_{MT} = \sum_{j=1}^{N_{dof}} \frac{\partial L_{MT}}{\partial q_j} \dot{q}_j \quad (3)$$

where V_{MT} is the muscle-tendon velocity and N_{dof} the number of degrees of freedom spanned by the muscle.

1.3 Ground contact model

Ground contact forces were simulated with a spring-damper based contact model. Contact geometry was modelled by one contact sphere at the heel and three at the metatarsal heads (Figure 1). Vertical reaction force F_y acting on each sphere was computed based on the indentation and indentation velocity of the contact sphere in the ground [4].

	r [m]	k $[Nm^{\frac{2}{3}}]$	c $\frac{s}{m}$
Heel	0.035	15	1
Toes	0.015	5	1

Supplementary Table 2: Parameters contact model

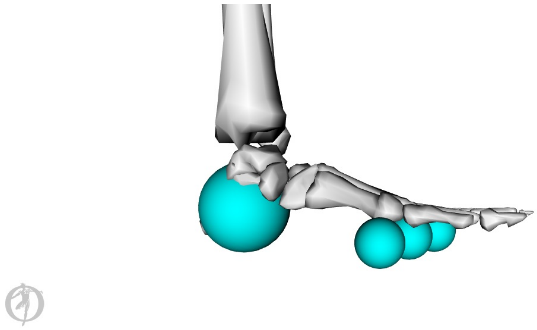

Supplementary Figure 1: Location of the four contact spheres on the foot

$$F_y = (10^6 k\sqrt{r}(y-r)^3)(1+c\dot{y}) \tag{4}$$

where k is the stiffness parameter, r is the radius of the sphere, y is the indentation of the sphere in the ground, c is the damping parameter and $\dot{y}$ is the indentation velocity of the sphere in the ground. Details on the contact parameters are shown in table 2 .
Friction forces (F_x and F_z) were computed as a function of the vertical ground reaction force and the horizontal velocity of the contact sphere.

$$F_x = F_y tanh(10\dot{x}) \tag{5}$$

$$F_z = F_y tanh(10\dot{z}) \tag{6}$$

where F_x and F_z is the friction force in respectively anterior-posterior and medio-lateral direction, $\dot{x}$ and $\dot{z}$ are respectively the velocity of the contact sphere in the anterior-posterior and medio-lateral direction.

2 Tracking skeletal dynamics

We created a model-based simulation of unperturbed walking by tracking the experimental data for a gait cycle of unperturbed walking. First, we solved for the joint torques that, when input to the skeleton dynamics, minimized the difference between simulated and measured movement (details below). Next, we solved for the muscle excitations that produced these joint torques by solving the muscle redundancy problem (see section 3).
We used the model described in section 1 and replaced the muscle-tendon actuators by joint actuators. Hence, model inputs were the joint torques and the model's state ($\boldsymbol{x}$) consisted of the generalized coordinates ($\boldsymbol{q}$) and velocities ($\dot{\boldsymbol{q}}$).

$$\boldsymbol{x} = [\boldsymbol{q} \quad \dot{\boldsymbol{q}}] \tag{7}$$

Model inputs (joint torques) were optimized to minimize the difference between the measured and simulated marker positions, ground reaction forces and joint torques:

$$J = \int_{t_0}^{t_{end}} \|(\boldsymbol{p} - \hat{\boldsymbol{p}}\|_2^2 + w_1 \sum_{j}^{nF} (\boldsymbol{F_j} - \hat{\boldsymbol{F}}_{\boldsymbol{j}})^2 + w_2 \|\boldsymbol{T} - \hat{\boldsymbol{T}}\|_2^2 dt \tag{8}$$

where t_0 is the time of left heelstrike and t_{end} the time of the consecutive left heelstrike, $\boldsymbol{p}$ is the vector containing the 3D marker coordinates of all 48 markers on the skeletal model, $\hat{\boldsymbol{p}}$ is a vector with the corresponding measured marker coordinates, $\boldsymbol{F}$ and $\hat{\boldsymbol{F}}$ are vectors containing respectively the simulated and measured ground contact forces and moments and nF equals two (ground reaction force under left and right foot). $\hat{\boldsymbol{T}}$ is a vector containing the inverse dynamic joint torques based on the measured kinematics and ground reaction forces, $\boldsymbol{T}$ is a vector containing the corresponding simulated joint torques.
The optimization was subject to skeleton dynamics. We solved this dynamic optimization problem using direct collocation. To improve the numerical condition of the resulting non-linear programming problem, we first reformulated the optimization problem as described below.

2.1 Problem formulation

First, we used an implicit formulation of skeleton dynamics [3]. To this aim, we introduced joint accelerations $\boldsymbol{u}_{\ddot{\boldsymbol{q}}}$ as additional controls, simplifying the dynamic equations:

$$\frac{d\dot{\boldsymbol{q}}}{dt} = \boldsymbol{u}_{\ddot{\boldsymbol{q}}} \tag{9}$$

Skeleton dynamics were then imposed by a path constraint:

$$\boldsymbol{T_{ID}} = f(\boldsymbol{q}, \dot{\boldsymbol{q}}, \boldsymbol{u}_{\ddot{\boldsymbol{q}}}) \tag{10}$$

Second, we defined the kinematic chain with the foot as a floating base, the position and orientation of the model was described by 6 degrees of freedom between the foot and the ground. By choosing the foot instead of the pelvis as a floating base, we obtained a more direct relation between the model's states (kinematics of the foot) and the ground reaction forces, which improved convergence of the optimization problem. As a consequence, we had to define seperate kinematic chains for the left and right leg, which were coupled at the pelvis [3]. Each chain had a reduced pelvis segment with a mass and inertia that equalled half of the mass and inertia of the original pelvis segment. We introduced forces $\boldsymbol{F_p}$ and moments $\boldsymbol{M_p}$ acting on the right reduced pelvis as additional controls. Reaction forces and moments were applied on the left reduced pelvis.
We then added additional path constraints to impose that the two reduced pelvises had the same kinematics. For both models, skeleton dynamics was evaluated through OpenSim's Inverse Dynamics Tool during the optimization. The residual forces and moments (i.e. external forces and moments) computed with inverse dynamics were constrained to be equal to the ground reaction forces computed with the contact model implemented in Matlab.

2.2 Summary optimal control problem

In summary, we solved the following dynamic optimization problem through direct collocation:

1. **State:**

$$\boldsymbol{x} = \begin{bmatrix} \boldsymbol{q} & \dot{\boldsymbol{q}} \end{bmatrix} \tag{11}$$

2. **Controls:**

$$u = \begin{bmatrix} u_{\ddot{q}} & F_p & M_p & T \end{bmatrix} \tag{12}$$

3. **Dynamic constraints:**

$$\dot{x} = \begin{bmatrix} \dot{q} & u_{\ddot{q}} \end{bmatrix} \tag{13}$$

4. **Path constraints:**

- Input joint torques should equal the inverse dynamics torques computed based on the model's kinematics.

$$\begin{bmatrix} T_{ID} & F_{ground-foot} \end{bmatrix} = f_{ID}(q, \dot{q}, u_{\ddot{q}}, F_p, M_p) \tag{14}$$

- Residual forces and moments at the foot from OpenSim's Inverse Dynamics Tool $F_{ground-foot}$ should equal the forces and moments computed based on the contact model.

$$F_{ground-foot} - F_{contact}(q, \dot{q}) = 0 \tag{15}$$

- position and orientation of the pelvis in the left and right model should be equal

$$P_{PelvisLeft}(q) = P_{PelvisRight}(q) \tag{16}$$

where F_p and M_p are the forces and torques to simulate the action of one model on the other, T_{ID} is a vector containing the inverse dynamics torques, $F_{ground-foot}$ is a vector containing the residual inverse dynamics forces and moments, $F_{contact}$ is vector containing the ground reaction forces and moments computed with the ground contact model, $P_{PelvisLeft}$ and $P_{PelvisRight}$ are vectors containing the position and orientation of the left and right pelvis in the ground.

2.3 Implementation

The direct collocation software GPOPS II was used to formulate the non-linear programming problem, which was solved using IPOPT. Inverse dynamics and the computation of the marker locations was solved using the OpenSim libraries trough a Mex file. Computation time was decreased by distributing the collocation points between multiple cores using OpenMPI. The ground contact forces and moments were computed in Matlab.

2.4 Tracking results

The root-mean square error of the tracking simulation of unperturbed walking was 0.03 cm for the marker positions, 29N for the ground reaction forces and 6.7 Nm for the joint torques. The tracking of the ground reaction forces (Figure 2) and joint torques (Figure 3) is shown for one representative trial.

3 Tracking muscle dynamics

In a second step, the muscle excitations that generate the inverse dynamic joint torques were computed using dynamic optimization. We modified the dynamic optimization approach proposed by [2] to include tracking of the gluteus medius activity (measured with electromyography). The objective function contains a part that minimizes muscle excitations squared, a second part the minimizes the differences between the measured and simulated excitations of the gluteus

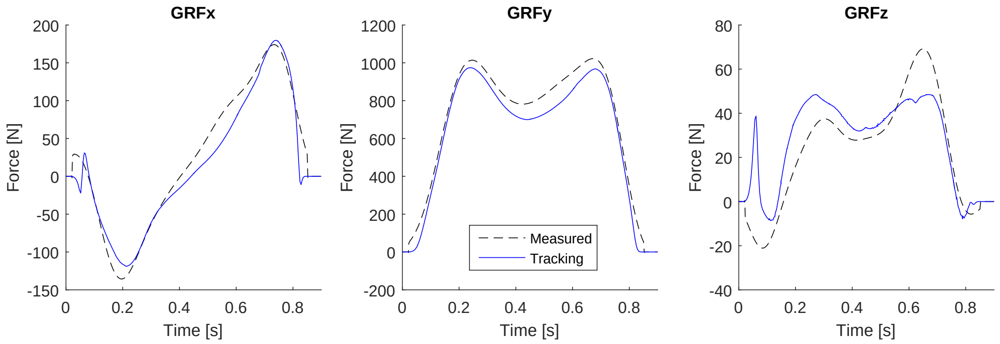

Supplementary Figure 2: Measured ground reaction forces (black dotted) versus simulated ground reaction forces (blue) for one representative trial of the tracking simulations.

medius and a third part that minimizes the torque generated by ideal torque actuators (i.e. reserve actuators according to OpenSim terminology) (Equation 17).

$$J = \int_{t_0}^{t_{end}} \sum_{i}^{nMus} e_i^2 + w_1(s\hat{e}_{GM} - e_{GM})^2 + w_2 \sum_{j}^{nDOF} e_{Tj} T_{max} dt \tag{17}$$

where nMus=92 is the number of muscles in the model, e_i is the simulated excitation of muscle i, $\hat{e}_{GM}$ is the processed EMG of the gluteus medius, s is an optimization variable and scales the EMG from voltage to muscle excitations, e_{GM} is the simulated excitation of the gluteus medius, $Tmax$ equals 150Nm and is the reserve actuator of degree of freedom j, e_{Tj} is the activation of the reserve actuator of DOF k. Note that the weight w_2 is high to penalize the use of the reserve actuators.
The reserve actuators were added to guarantee feasibility in the presence of fast changes in joint torques in the tracking simulation that can not be generated by the muscles.This objective function was minimized subject to dynamic constraints following from muscle activation and contraction dynamics and to path constraints imposing that the model's actuators (muscles, passive torque, reserve actuators) should produce the inverse dynamics joint torques. In this case, the inverse dynamics joint torques were the result of the tracking simulation described in section 2. Reserve actuator torques did not exceed 2 Nm in the simulation. Joint torques resulting from the muscles, passive forces and reserve actuators were imposed to be equal to the joint torques from tracking the skeletal dynamics. The tracking of the gluteus medius activity in one representative trail can be found in Figure 4.
The source code of the muscle redundancy solver can be found on the simtk project optcntrlmuscle (`https://simtk.org/projects/optcntrlmuscle`).

4 Forward simulation

The initial state and simulated muscle excitations from the tracking simulations were used to reconstruct the unperturbed walking in a forward simulation. The passive response to the perturbation was simulated by imposing the platform translation as an external force in the forward simulation of unperturbed walking. The active response was simulated by imposing the perturbation force and adding the measured muscle response to the perturbation (i.e. the difference between measured activity during unperturbed walking and after perturbation) to the muscle excitations from the tracking simulation of unperturbed walking. Muscle and skeletal

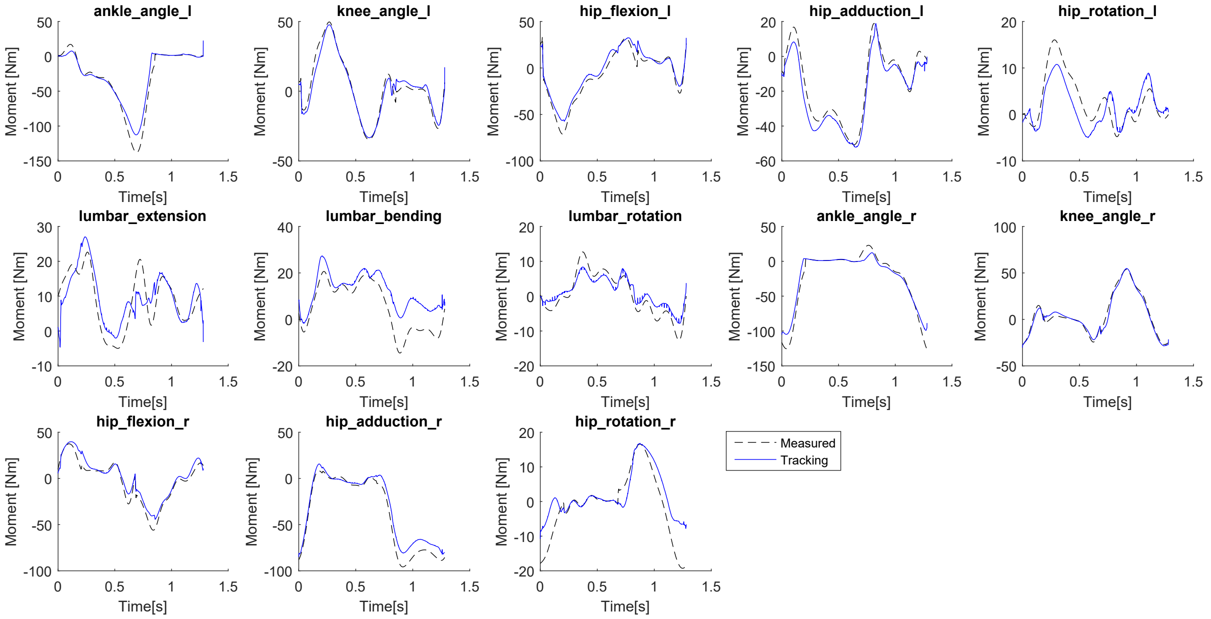

Supplementary Figure 3: Estimated joint torques from the measured ground reaction forces and kinematics with inverse dynamics (black dotted) versus simulated joint torques (blue) for one representative trial of the tracking simulations.

dynamics were evaluated implicitly and integrated with a fully implicit integrator in Matlab (ode15i, absolute tolerance 10^{-5}). A time scaling factor (w) of 10^4 was used to improve the numerical condition (Equation 18)).

$$\tilde{t} = wt \tag{18}$$

$$\tilde{\boldsymbol{q}} = \boldsymbol{q} \tag{19}$$

$$\dot{\tilde{\boldsymbol{q}}} = \frac{\dot{\boldsymbol{q}}}{w} \tag{20}$$

$$\ddot{\tilde{\boldsymbol{q}}} = \frac{\ddot{\boldsymbol{q}}}{q^2} \tag{21}$$

5 Sensitivity analysis

5.1 Stiffness contact model

The sensitivity of the simulated active and passive response to contact model properties was evaluated by modifying the stiffness of the contact model in the tracking and forward simulation. The stiffness k was stepwise increased in the tracking simulation of one representative trial (Equation 4). The results from the tracking simulation and the modified contact model were used in the forward integration of the passive and active response. The stiffness had only a small influence on the simulated ground reaction forces in the tracking simulation and had only a limited effect on the simulated stride width and margin of stability in the active and passive response (Figure 5). Since the contact stiffness had only a small influence on the simulation results, we selected a relatively low stiffness value $k = 10$ to decrease the stiffness of the differential equations.

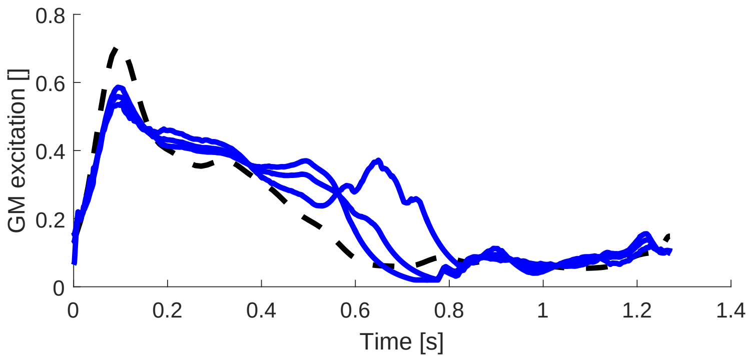

Supplementary Figure 4: Measured (black dotted) and simulated activity of the bundles of the gluteus medius (blue) in one representative trial.

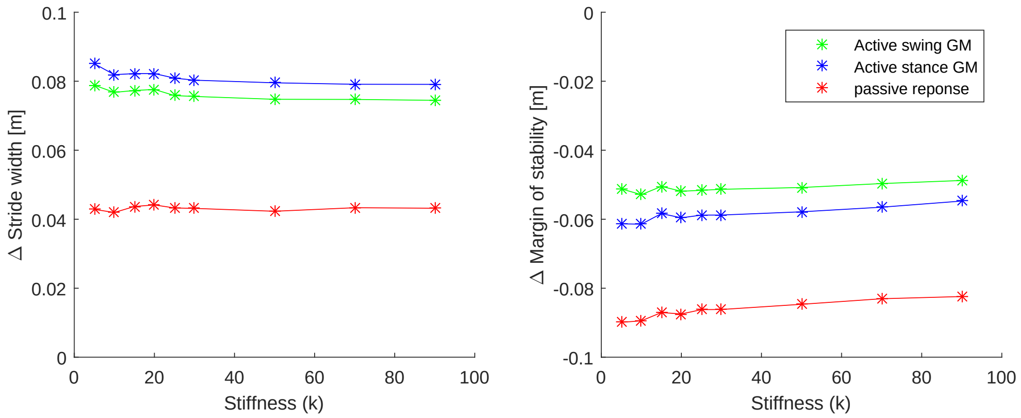

Supplementary Figure 5: Influence stiffness of the contact model on stride width and margin of stability in the simulated active (blue and green) and passive (red) response to the perturbation.

5.2 Lower bounds on activity

It has been hypothesized that muscle co-contraction is used as a strategy to control balance during walking by increasing the joint stiffness [5]. The influence of this co-contraction strategy on the simulated passive response was evaluated by constraining the minimal muscle activity in the muscle redundancy solver. The minimal muscle activity was increased stepwise in the muscle redundancy solver. The stride width and margin of stability at first heelstrike after perturbation was computed in the passive and active response using forward simulations for the different levels of minimal muscle activity. Stride width decreased with increased muscle activity and a small change in the margin of stability was found in the active and passive response simulations (Figure 6).

6 Measurements

6.1 Heelstrike detection

Left and right heel strikes were determined from the ground reaction force. When the subjects were walking with one foot on each belt, a simple vertical force threshold of 30N was used to

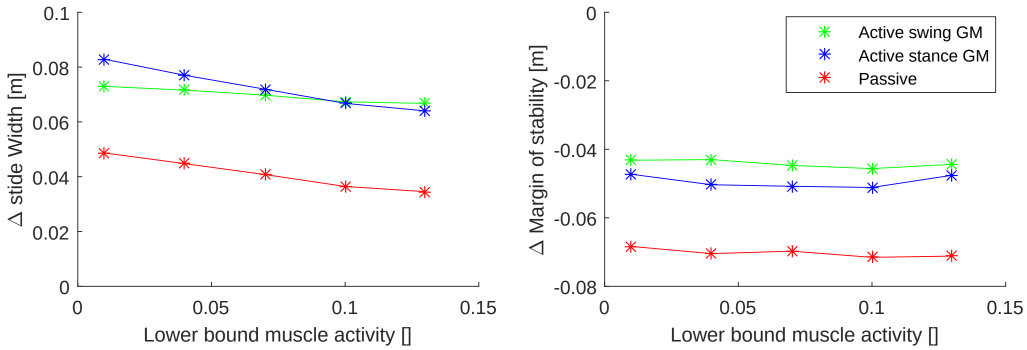

[H]

Supplementary Figure 6: Influence of lower bound on muscle activity on stride width and margin of stability in the simulated active (blue and green) and passive (red) response to the perturbation.

detect initial contact of the foot with the ground. An additional criteria was needed to detect heelstrikes when the perturbation caused an inward stepping strategy (subjects were therefore walking with both feet on one belt). Peaks in anterior-posterior movement of the combined center of pressure were identified using Matlab's peakfind function and were used to detect heelstrikes (Figure 7). The difference between the center of pressure based and vertical force threshold is on average 0.007s for normal split belt treadmill walking (standard deviation equals 0.002s).

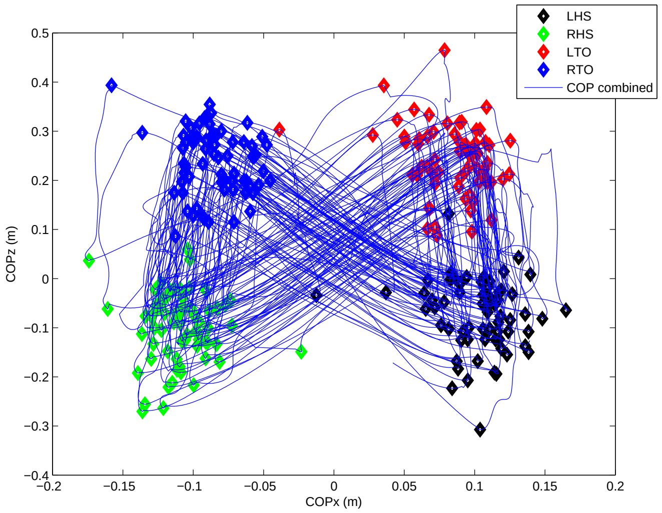

Supplementary Figure 7: Peaks in anterior-posterior movement of the combined center of pressure (blue line) were used to detect heelstrikes when subjects were walking with both feet on one belt. The black and green dots are the peak in the anterior-posterior movement of the combined center of pressure and represent respectively the left heelstrike (lhs) and right heelstrike (rhs). The red and blue dots represent respectively the left toe-off (lto) and right toe-off (rto).

6.2 Marker protocol

Whole-body motion was recorded with an extended plug in gait marker set (figure 8).

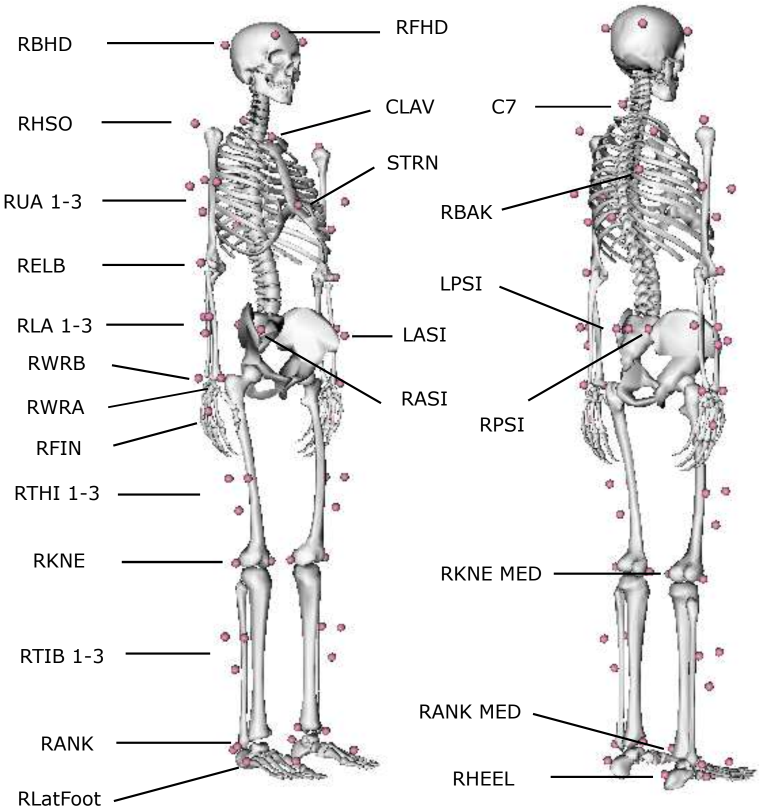

Supplementary Figure 8: Motion capture markers visualised on the musculoskeletal model used in the inverse analysis

Head markers

RFHD	Right front head	Located approximatly over the right temple
RBHD	Right back head	Placed on the back of the head

Torso markers

C7	7th Cervical vertebrae	Spinous process of the 7th cervical vertebrae
T10	10th Thoracic Vertebrae	Spinous Process of the 10th thoracic vertebrae
CLAV	Clavicle	Jugular notch where the clavicles meet the sternum
STRN	Sternum	Xiphoid process of the Sternum
RBAK	Right Back	Placed in the middle of the right scapula

Arm Markers

RHSO	Right shoulder marker	Placed on the acromio-clavicular joint
RUA 1-3	Right upper arm cluster	Upper arm between the elbow and shoulder marker
RELB	Right elbow	Lateral epicondyle
RLA 1-3	Right lower arm cluster	Lower arm between the elbow and wrist
RWRA	right wrist	Right wrist bar thumb side
RWRB	right wrist	Right wrist bar pinkie side
LFIN	Left fingers	Dorsum of the hand below the head of the second metacarpal

Pelvis

RASI	Right ASIS	Right anterior superior iliac spine
RPSI	Right PSIS	Right posterior superior iliac spline

Leg Markers

RTHI 1-3	Right femur cluster	Lower lateral surface of the thigh
RKNE	Right knee	Lateral epicondyle of the right knee
RKNE MED	Right knee	Medial epicondyle of the right knee
LTIB 1-3	Right tibial cluster	Lateral side of the shank
RANK	Right ankle	Lateral malleolus
RANK MED	Right ankle	Medial malleolus
RLatFoot	Right foot	tuberositas ossis metatarsi

References

[1] Frank C. Anderson and Marcus G. Pandy. Dynamic Optimization of Human Walking. *Journal of Biomechanical Engineering*, 123(5):381, oct 2001.

[2] Friedl De Groote, Allison L. Kinney, Anil V. Rao, and Benjamin J. Fregly. Evaluation of Direct Collocation Optimal Control Problem Formulations for Solving the Muscle Redundancy Problem. *Annals of Biomedical Engineering*, 44(10):2922–2936, oct 2016.

[3] Andrew J. Meyer, Ilan Eskinazi, Jennifer N. Jackson, Anil V. Rao, Carolynn Patten, and Benjamin J. Fregly. Muscle Synergies Facilitate Computational Prediction of Subject-Specific Walking Motions. *Frontiers in Bioengineering and Biotechnology*, 4:77, oct 2016.

[4] Michael A Sherman, Ajay Seth, and Scott L Delp. Simbody: Multibody dynamics for biomedical research. In *Procedia IUTAM*, volume 2, pages 241–261, 2011.

[5] Heather E Stokes, Jessica D Thompson, and Jason R Franz. The Neuromuscular Origins of Kinematic Variability during Perturbed Walking. *Scientific Reports*, 7(1), 2017.

[6] Antonie J. van den Bogert, Thomas Geijtenbeek, Oshri Even-Zohar, Frans Steenbrink, and Elizabeth C. Hardin. A real-time system for biomechanical analysis of human movement and muscle function. *Medical & Biological Engineering & Computing*, 51(10):1069–1077, oct 2013.
